# Carbyne-Enriched Carbon Coatings on Silicon Chips as Biosensing Surfaces with Stable-over-Time Biomolecule Binding Capacity

**DOI:** 10.3390/nano15181384

**Published:** 2025-09-09

**Authors:** Dimitra Tsounidi, Panagiota Petrou, Mariya Aleksandrova, Tsvetozar Tsanev, Angeliki Tserepi, Evangelos Gogolides, Andrzej Bernasik, Kamil Awsiuk, Natalia Janiszewska, Andrzej Budkowski, Ioannis Raptis

**Affiliations:** 1 Immunoassays/Immunosensors Lab, Institute of Nuclear and Radiological Sciences and Technology, Energy and Safety, National Centre For Scientific Research Demokritos, 15341 Aghia Paraskevi, Greece; dimitratsounidi@gmail.com; 2 Department of Microelectronics, Technical University of Sofia, 1000 Sofia, Bulgaria; m_aleksandrova@tu-sofia.bg (M.A.); zartsanev@tu-sofia.bg (T.T.); 3 Institute of Nanoscience and Nanotechnology, National Centre For Scientific Research Demokritos, 15341 Aghia Paraskevi, Greece; a.tserepi@inn.demokritos.gr (A.T.); e.gogolides@inn.demokritos.gr (E.G.); i.raptis@inn.demokritos.gr (I.R.); 4 Faculty of Physics and Applied Computer Science, AGH University of Krakow, 30-059 Kraków, Poland; bernasik@agh.edu.pl; 5 Marian Smoluchowski Institute of Physics, Faculty of Physics, Astronomy and Applied Computer Science, Jagiellonian University, 30-348 Kraków, Poland; kamil.awsiuk@uj.edu.pl (K.A.); natalia.janiszewska@doctoral.uj.edu.pl (N.J.); andrzej.budkowski@uj.edu.pl (A.B.)

**Keywords:** carbyne-enriched, carbon coatings, biosensors, White Light Reflectance Spectroscopy, amine–biotin derivative, C-reactive protein

## Abstract

Carbyne-containing materials offer significant potential for biosensor applications due to their unique chemical and mechanical properties. In this study, carbyne-enriched carbon coatings deposited on SiO_2_/Si chips using ion-assisted pulse-plasma deposition were evaluated for the first time as substrates for optical biosensing. At first, the carbyne-enriched coatings were characterized by X-ray photoelectron spectroscopy, Raman spectroscopy, Atomic Force Microscopy, and the sessile drop method to assess their composition, structure, and wettability. After that, chips with carbyne-enriched coatings were modified with biomolecules through physical absorption or covalent bonding, and the respective biomolecular interactions were monitored in real-time by White Light Reflectance Spectroscopy (WLRS). In both cases, SiO_2_/Si chips modified with an aminosilane were used as reference substrates. Physical adsorption was tested through immobilization of an antibody against C-reactive protein (CRP) to enable its immunochemical detection, whereas covalent bonding was tested through coupling of biotin and monitoring its reaction with streptavidin. It was found that the carbyne-enriched carbon-coated chips retained both their antibody adsorption capability and their covalent bonding ability for over 18 months, while the modified with aminosilane SiO_2_/Si chips lost 90% of their antibody adsorption capacity and covalent bonding ability after two months of storage. These findings highlight the strong potential of carbyne-enriched carbon-coated chips as robust biosensing substrates, with applications extending beyond WLRS.

## 1. Introduction

The immobilization of biomolecules on solid surfaces is a critical step in the development of biosensors as it directly impacts the sensitivity, specificity, and repeatability of the sensor’s responses [1]. Silicon-based substrates provide a versatile platform for sensor fabrication and integration into devices. However, achieving consistent and reproducible biomolecule immobilization on silicon-based surfaces requires chemical activation, which complicates the biosensor fabrication process and introduces response variability [2,3,4]. To overcome these challenges, the coating of silicon surfaces with a functional intermediate layer between the substrate and the biomolecules has emerged as a promising approach to enable efficient, stable, and repeatable biomolecule immobilization without the need for extensive chemical activation steps [5,6].

Over the past few decades, carbon nanomaterials have attracted significant attention and extensive investigation for their applications in biosensor development owing to their remarkable physical and chemical properties. Among the various carbon allotropes, fullerenes, carbon nanotubes, graphene, and nanodiamonds stand out as the most widely used materials in the field of biosensors. Materials composed of zero-dimensional (fullerenes), one-dimensional (carbon nanotubes), two-dimensional sp^2^-hybridized carbon atoms (graphene), and three-dimensional sp^3^-hybridized carbon atoms (nanodiamonds) have been employed as transducer surfaces for the immobilization of biorecognition molecules, capitalizing on their high surface-to-volume ratio, biocompatibility, and functionalization capabilities [5,7,8]. Furthermore, these materials can be integrated with several signal transduction principles, including fluorescence, surface plasmon resonance (SPR), field-effect transistors (FET), and electrochemical sensing [7,8,9,10,11].

Among the various known carbon allotropes, carbyne has a unique form, being composed of one-dimensional sp-hybridized carbon atoms [12]. Carbyne has exceptional mechanical properties, possessing twice the stiffness of other carbon materials [13,14] and unique optical and electrical characteristics, including semiconducting behavior and chemical stability [13,14,15,16]. These characteristics have sparked significant interest in carbyne across various technological fields, including the field of sensors. Thus, several studies have demonstrated its potential applications in optoelectronic devices due to its luminescence properties [12], in the development of chemical gas sensors [17,18,19], and as an enzyme mimetic [20]. Carbyne nanocrystals have also been identified as promising probes for fluorometric and colorimetric ion detection [21]. However, reports of optical carbyne-based biosensors have not yet appeared in the literature, despite the advantages such a layer could offer with respect to biorecognition molecule attachment.

In this study, silicon dioxide/silicon (SiO_2_/Si) chips with two types of carbyne-enriched carbon coatings were evaluated as substrates for biomolecule detection by an optical biosensor based on White Light Reflectance Spectroscopy (WLRS). WLRS has proven to be a robust and effective optical detection method for the rapid and accurate detection of various analytes in both biological [22,23] and food matrices [22,24], as well as for the evaluation of the efficiency of biomolecule immobilization methods [25,26]. The operational principle of the WLRS biosensor is based on the label-free monitoring of immunoreactions on biofunctionalized SiO_2_/Si chips by observing shifts in the interference spectrum created when white light reflects off the silicon chip through the silicon dioxide and the accumulating biomolecular adlayer [22]. The SiO_2_/Si chips with the carbyne-enriched carbon coatings used in this study were prepared using ion-assisted pulse-plasma deposition [27]. Prior to their implementation as biosensing surfaces, the carbyne-enriched carbon layers were characterized using various surface science techniques, including X-ray photoelectron spectroscopy (XPS), Raman spectroscopy and microscopy, Atomic Force Microscopy (AFM), and the sessile drop method to determine their composition, surface structure, and wettability. The composition of the carbyne-enriched carbon layers was compared, based on the literature data, with those formed following different methods, including laser ablation techniques [28,29], dehydrohalogenation of polyvinyl chloride and polyvinylidene chloride copolymer [30], carbon plasma [31], or RF magnetron sputtering [32]. Following their physicochemical characterization, the performance of the SiO_2_/Si substrates with a carbyne-enriched carbon coating as WLRS biosensing chips was evaluated after modification with biomolecules either by covalent bonding or physical adsorption. To test covalent bonding, an amine-terminated biotin derivative was used, and the efficiency of the reaction was evaluated through monitoring of immobilized biotin moieties’ interaction with streptavidin. Physical adsorption, on the other hand, was tested through the immobilization onto the chips of an affinity-purified goat polyclonal antibody against C-reactive protein (CRP), a well-established biomarker for inflammation [33,34,35]. The antibody-coated chips were then used for the immunochemical determination of CRP through a two-step non-competitive immunoassay. The chips with carbyne-enriched carbon coatings were compared to aminosilane-modified SiO_2_/Si chips with respect to their biomolecule immobilization capacity, both through covalent bonding and physical adsorption, as well as regarding the stability of their immobilization properties over time. Since modification with APTES is the most widely employed method for chemical activation of silicon-based optical biosensors [36], the findings demonstrate the great potential of chips with carbyne-enriched carbon coating as appropriate or superior substrates for biomolecule immobilization for biosensing applications.

## 2. Materials and Methods

### 2.1. Materials

Affinity-purified goat antibody against human C-reactive protein (code GC019) and C-reactive protein (CRP) from human fluids were purchased from Scripps Laboratories (San Diego, CA, USA). Bovine serum albumin (BSA) and (3-aminopropyl)triethoxysilane (APTES) were purchased from Acros Organics (Geel, Belgium). EZ-Link™ Amine-PEG3-Biotin (amine-terminated biotin), sulfo-N-hydroxysulfosuccinimide (sulfo-NHS), bis(sulfosuccinimidyl) suberate (BS3), and streptavidin were purchased from Thermo Fisher Scientific Inc. (Waltham, MO, USA). N-(3-Dimethylaminopropyl)-N′-ethylcarbodiimide hydrochloride (EDC), 2-(N-morpholino)ethanesulfonic acid (MES), and all other reagents were purchased from Merck (Darmstadt, Germany). The water used throughout this study was distilled. Four-inch device-quality Si wafers were purchased from Si-Mat Germany (Kaufering, Germany). A silicon dioxide layer with 1000 nm average thickness was grown on the Si wafers through thermal oxidization at 1100 °C for 4 h at the Nanotechnology and MEMS lab of the Institute of Nanoscience and Nanotechnology of NCSR “Demokritos”. Then, the wafers were diced into chips with dimensions of 5 mm × 15 mm to fit in the docking station of the WLRS biosensing platform.

### 2.2. WLRS Instrumentation

**Figure 1:**
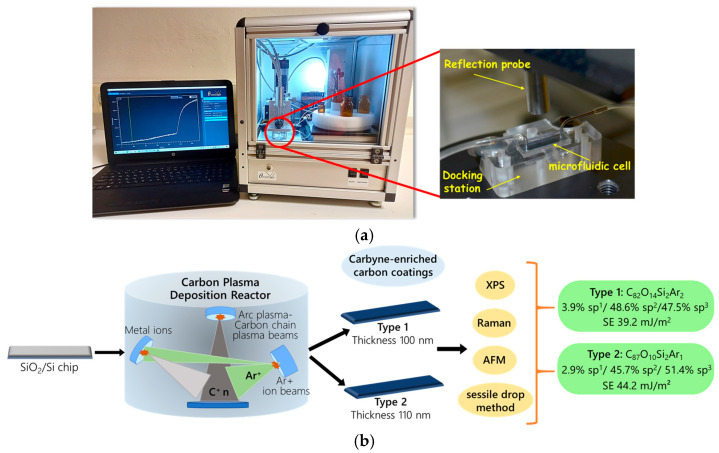
(**a**) Image of the WLRS-based biosensor instrument used in this study; (**b**) schematic depiction of the procedure for preparation and characterization of Type 1 and Type 2 carbyne-enriched carbon coatings onto the SiO_2_/Si chips.

The optical setup of the WLRS biosensor (ThetaMetrisis SA., Athens, Greece) includes a light source emitting in the visible/near-infrared spectral range, a miniaturized spectrometer (Maya 2000 Pro, 16-bit A/D; Ocean Optics, Duiven, The Netherlands) that operates in the 450–650 nm spectral range, and a reflection probe consisting of six illumination fibers—each with a core diameter of 400 μm—and one collecting fiber of the same diameter that guides the reflected light to the spectrometer. The biofunctionalized chip is combined with a custom-designed microfluidic cell (Jobst Technologies GmbH, Freiburg, Germany) for reagent delivery and is placed in a docking station that ensures automatic alignment with the reflection probe. The microfluidic cell is connected to a rotating sampler and a peristaltic micropump, both programmable through dedicated software to automatically execute the immunoassay protocol [23]. An image of the reader, along with the PC running the software (FR-Scanner, v2025.5.29.1020; ThetaMetrisis SA., Athens, Greece), is provided in Figure 1a. During the assay, the software collects and analyzes the reflectance spectrum from the biochip at a rate of one spectrum per second, providing real-time monitoring of the effective biomolecular adlayer thickness formed on the biochip throughout the immunoassay. A detailed description of the mathematical processing of the reflectance spectra collected during the immunoassay to calculate the effective biomolecular adlayer thickness in nm is provided in the Supplementary Materials. Based on the thickness values obtained for each one of the calibrators, a calibration curve was constructed. The corresponding linear regression equation was then loaded into the software and used to calculate the analyte concentration in the samples.

### 2.3. Deposition and Characterization of Carbyne-Enriched Carbon Layer on SiO_2_/Si Chips

Two different types of carbyne-enriched carbon layers were deposited on SiO_2_/Si substrates at SWISSIMPIANTI Sagl (Balerna, Switzerland) using arc-pulse carbon plasma technology. The first one was deposited using the following plasma parameters: a 1 m target–substrate distance; 7000 carbon plasma pulses; voltage difference between the graphite cylindrical main discharge cathode holding the carbon source and the main discharge anode holding the substrate at 300 V. The main capacitor charge was 2000 μF at 3 Hz, and the ion plasma assistance of Ar was 750 V at 70 mA. The layer created under these conditions had a thickness of 100 nm (Type 1). Changing the carbon plasma pulses to 8000 and the voltage difference between the main discharge cathode and anode to 200 V, a 110-nanometer-thick carbyne-enriched carbon layer (Type 2) was deposited on the SiO_2_/Si substrates.

The carbyne-enriched carbon layers were characterized by X-ray photoelectron spectroscopy (XPS), Raman spectroscopy and microscopy, Atomic Force Microscopy (AFM), and water contact angle measurements. XPS measurements were carried out using a PHI VersaProbe II Scanning XPS system (Physical Electronics, Inc., Chanhassen, MN, USA), with monochromatic Al Kα (1486.6 eV) X-ray source focused on a 100 µm spot and scanned over an area of 400 × 400 µm^2^. The photoelectron take-off angle was 45°. The pass energy in the analyzer was set to 117.50 eV (0.5 eV step) for survey scans and 46.95 eV (0.1 eV step) to obtain high-resolution spectra. Dual-beam charge compensation with 7 eV Ar^+^ ions and 1 eV electrons was used. The samples were also sputtered (over an area of 3 × 3 mm^2^) for 5 min using the Argon gas cluster ion beam (Ar-GCIB) (10 kV beam voltage, 28 nA current, with approximately 4000 atoms per cluster) applying Zalar rotation. All XPS spectra were referenced to the argon at the graphite peak (Ar 2p, 241.5 eV). The analytical chamber pressure was <3 × 10^−9^ mbar. The spectra were analyzed with the PHI MultiPak software (v.9.9.3). The background spectrum was subtracted using the Shirley method. Raman spectroscopy and microscopy data were obtained with a confocal Raman microscope (WITec alpha300 RAS, WITec, GmbH, Ulm, Germany) equipped with a 532 nm laser light source (10.2 mW power output) and 600 groove/mm grating, with spectra collected from the entire imaged areas of 14 × 14 µm^2^. The samples were measured after their immersion in water to reduce the impact of the exciting laser light power. AFM images were recorded using a WITec alpha300 RAS microscope working in non-contact mode, equipped with silicon probes with a spring constant of about 2.8 N/m and resonant frequencies of about 75 kHz. The root-mean-square (RMS) measure, calculated using Gwyddion software (version 2.65) [37], was taken as a surface roughness. Contact angle and surface energy data were acquired by the sessile drop technique using a Kruss EasyDrop instrument (DSA15; A. Krüss Optronic GmbH, Hamburg, Germany) at room temperature. Static contact angles for water and diiodomethane droplets (at least 7) were determined. Then, the Owens−Wendt−Kaelble analytical approach was applied to calculate the surface free energy and its dispersion and polar components [38]. The procedure followed for the preparation and characterization of Type 1 and Type 2 carbyne-enriched carbon coatings is schematically depicted in Figure 1b.

### 2.4. Modification of SiO_2_/Si Chips with APTES

The SiO_2_/Si chips were cleaned by successive sonication for 10 min in baths of acetone and 2-propanol, followed by drying under a nitrogen flow. Then, the chips were immersed for 20 min in Piranha solution (1:1 *v*/*v* H_2_SO_4_/30% H_2_O_2_) for surface cleaning and hydrophilization, followed by extensive washing with distilled water and drying under nitrogen flow. The cleaned chips were then activated with 3-aminopropyl-triethoxysilane (APTES) through immersion for 20 min in a 2% (*v*/*v*) aqueous APTES solution, followed by gentle washing with distilled water and drying with nitrogen. The chips were cured at 120 °C for 20 min, and kept at room temperature (RT) in a desiccator.

### 2.5. Immobilization of Anti-CRP Antibody and WLRS CRP Assay

For the immobilization of the anti-CRP antibody onto SiO_2_/Si chips modified with a carbyne-enriched carbon layer or APTES, a 3 × 5 mm^2^ area at the center of each chip was spotted with a 100 μg/mL anti-CRP antibody solution in 0.05 M carbonate buffer, pH 9.2, using the BioOdyssey Calligrapher MiniArrayer (Bio-Rad Laboratories Inc., Hercules, CA, USA). To cover the designed area, 15 × 25 spots of the antibody solution with a mean diameter of 350 μm, each corresponding to a volume of 12 nL, were deposited with a spot-to-spot distance of 200 μm, leading to a total volume of antibody solution per chip of approximately 4.5 μL. After overnight incubation at RT in a humidity chamber (75% humidity), a blocking step was performed by immersing the biofunctionalized chips in a Petri dish containing 10 mL of blocking solution (1% *w*/*v* BSA in 0.1 M NaHCO_3_, pH 8.5) for 2 h at RT. The biofunctionalized chips were then washed with washing solution (0.01 M Tris-HCl, pH 8.5), dried with nitrogen, and stored at 4 °C until use.

Prior to the assay, each chip was assembled with the microfluidic cell and placed in the docking station of the WLRS instrument, ensuring proper connections to the micropump and the reagent handling module. The chip was then equilibrated with assay buffer (0.05 M Tris-HCl, pH 7.8, 0.9% *w*/*v* NaCl, 0.5% *w*/*v* BSA). CRP calibrators prepared in the assay buffer were subsequently run over the chip for 7 min at a flow rate of 40 μL/min, followed by a 5 μg/mL anti-CRP antibody solution in assay buffer for 5 min at the same flow rate. A regeneration step was then performed by running a 0.1 M glycine HCl solution, pH 2.5, over the chip for 3 min, followed by re-equilibration with assay buffer before the next immunoassay cycle. The assay procedure is depicted schematically in Figure S1.

### 2.6. Covalent Bonding of Amine-Terminated Biotin onto APTES-Modified Chips

For the covalent immobilization of amine-terminated biotin on the APTES-modified SiO_2_/Si chips, chemical activation of the amine groups was performed through incubation with 100 μL of a 1 mg/mL solution of BS3 in 0.1 M PBS buffer, pH 7.4, for 30 min at RT. After washing with the same buffer, the chips were incubated with 100 μL of a 1 mg/mL amine-PEG_2_-biotin solution in 0.1 M PBS buffer, pH 7.4, for 45 min at RT. The biofunctionalized chips were washed and then immersed in a Petri dish containing 10 mL of blocking solution for 30 min at RT and, finally, washed with distilled water and dried with nitrogen. After that, the chips were assembled with the microfluidic cell and equilibrated with 0.1 M PBS buffer, pH 7.4, 0.5% *w*/*v* BSA, prior to running for 5 min a 2 μg/mL streptavidin solution in the same buffer. Finally, the buffer was run over the chips to wash out streptavidin.

### 2.7. Covalent Bonding of Amine-Terminated Biotin onto Chips with Carbyne-Enriched Carbon Coatings

For the covalent immobilization of amine-terminated biotin on the SiO_2_/Si chips with the carbyne-enriched carbon layer, chemical activation of the carboxyl groups was performed through incubation with 100 μL of a mixture containing 10 mM EDC and 5 mM sulfo-NHS in 0.1 M MES buffer, pH 5.5, for 1 h at RT. After washing, the chips were incubated with 100 μL of a 1 mg/mL amine-PEG2-biotin solution in 0.1 M PBS buffer, pH 7.4, for 45 min at RT. The biofunctionalized chips were washed, blocked, and tested as described in Section 2.6 for the APTES-modified SiO_2_/Si chips.

## 3. Results and Discussion

### 3.1. Characterization of Carbyne-Enriched Carbon Layers Deposited onto SiO_2_/Si Chips

**Figure 2:**
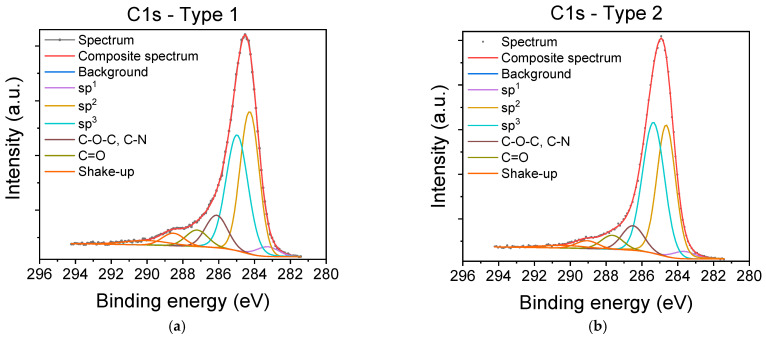
High-resolution C1s core-level XPS spectra of (**a**) Type 1 and (**b**) Type 2 carbyne-enriched carbon coatings (analyzed with sampling depth of 6.2 nm). Apart from the shake-up satellites, distinct contributions from different chemical bonds (C=O, C-O/C-N) and hybridizations (sp^1^, sp^2^, sp^3^) are visible.

The chemical composition, chemical states, and carbon hybridization of carbyne-enriched carbon layers deposited onto SiO_2_/Si chips using arc-pulse carbon plasma were evaluated by X-ray photoelectron spectroscopy (XPS). In particular, the high-resolution C1s core-level XPS spectra (Figure 2) revealed carbon chemical bonds and sp^n^ hybridizations. In addition to the shake-up satellites (centered around 288.9 and 290.2 eV) and the peaks corresponding to C=O (~287.6 eV) and C-O/C-N moieties (~286.3 eV), the spectra also show contributions from sp^3^-bonded (~285.1 eV), sp^2^-bonded (~284.4 eV), and sp^1^-bonded carbon (~283.4 eV) [14]. These contributions to the C1s spectra are similar for both Type 1 and Type 2 carbyne-enriched carbon coatings (Figure 2a and Figure 2b, respectively). As a result, the surfaces of both coatings are alike in their distributions of carbon hybridization states (see Table S1), with 3.9% of sp^1^, 48.6% of sp^2^, and 47.5% of sp^3^-bonded carbons for Type 1 films, and 2.9% of sp^1^, 45.7% of sp^2^, and 51.4% of sp^3^-bonded carbons for Type 2 coatings. Comparable non-zero (3.1–5.2%, Table S1) contributions of sp^1^ to carbon hybridization states were previously reported using XPS for various carbyne-containing carbon coatings [32,33,34,35]. Interestingly, carbyne-rich films synthesized with carbon plasma technology show a distribution of sp^n^ hybridization states similar to that of the Type 1 and Type 2 coatings (Table S1). Since XPS is a surface-sensitive technique (with a sampling depth below 10 nm), both coatings were re-examined after sputtering for 5 min with an Argon gas cluster ion beam. The evaluated compositions of the surface and interior of both types of carbyne-enriched carbon coatings, along with those of the surface reference SiO_2_, are presented in Figure S2. The surfaces of both coatings contain more oxygen and less carbon than their interiors, as expected, and the differences in elemental composition between Type 1 and Type 2 coatings are not larger than those variations, with 81.8–94.0 at% carbon and 3.3–13.5 at% oxygen. The bonding environments of carbon atoms are affected by their sp^n^ hybridization state (78.0–85.0%), and also by C=O (4.0–5.0%) and C-O/C-N bonds (8.0–10.0%). Similar XPS observations have been previously reported for other films containing carbyne [30,31,32]. In addition, excitations of delocalized π-electrons during photoemission in some carbon atoms (4.0–7.0%) are the most probable cause of shake-up satellites, as reported for graphitic, graphene, and carbon nanotube materials [39]. Finally, the presence of adventitious carbon contamination with sp^3^-bonded carbon (9.1 at%) on the surface of the samples is revealed by the SiO_2_ data. Taking this effect into account, the relative ratio of sp^1^-coordinated carbyne species to sp^2^- and sp^3^-bonded carbon components sp^1^/(sp^2^ + sp^3^) is evaluated as equal to 4.8% and 3.5% for the surfaces of the Type 1 and Type 2 coatings, respectively.

The ratio sp^1^/(sp^2^ + sp^3^) can also be estimated from Raman spectroscopy as the intensity ratio of the carbyne band to the sum of the D and G bands I_carbyne_/(I_D_ + I_G_) [40]. Even a small value of the relative peak intensity of carbyne I_carbyne_/(I_D_ + I_G_) can suggest a substantial initial concentration of carbyne, as has been indicated in previous publications [41,42]. In these reports, a high carbyne peak intensity, I_carbyne_/(I_D_ + I_G_) of 45%, for the carbyne-enriched carbon layer just deposited under UHV was observed followed by a rapid exponential decay (with a time constant of 0.58(3) h) after exposure to dry air, resulting in an I_carbyne_/(I_D_ + I_G_) value of 3.6(2)%, corresponding to their earlier ex situ Raman measurements [43]. Therefore, the small but non-zero ratio sp^1^/(sp^2^ + sp^3^), determined by ex situ XPS spectroscopy (Table S1), indicates a higher initial concentration of carbyne species.

**Figure 3:**
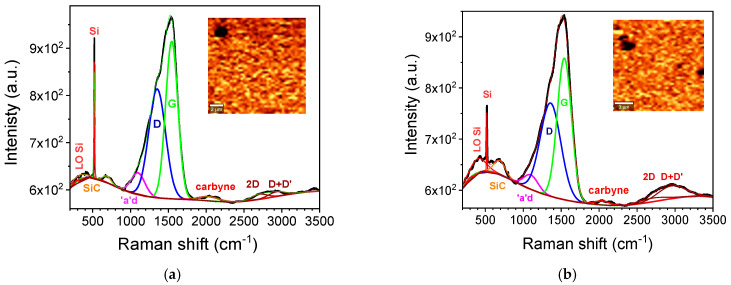
Raman micro-spectroscopic analysis of (**a**) Type 1 and (**b**) Type 2 carbyne-enriched coatings. Spectra collected with a micrometer sampling depth from 14 × 14 µm^2^ areas are depicted as intensity maps in the insets, revealing contributions characteristic of SiO_2_/Si substrate, sp^2^- and sp^3^-bonded carbon allotropes, and carbyne.

The examination of the samples with Raman microscopy revealed details of the coating morphology, chemical composition, and carbon hybridization. Raman scattering spectra (Figure 3), collected with micrometer-scale sampling depth from the areas depicted as intensity maps (see insets), revealed several characteristic contributions from the SiO_2_ substrate (silicon and silicon carbide), sp^2^- and sp^3^-bonded carbon allotropes, and carbyne. In particular, the silicon peak at 520 cm^−1^ is accompanied by bands centered at 400 cm^−1^ due to the Si-Si longitudinal optical mode, and at 690 cm^−1^ due to the stretching of Si–C bonds [44]. The components of the spectra related to sp^2^- and sp^3^-bonded carbon begin with the minor band located at 1100 cm^−1^, typically ascribed to an “amorphous” diamond [45]. The peak centered at 1350 cm^−1^ corresponds to the “disorder” D-band of disordered polycrystalline graphite, which is close to the 1332 cm^−1^ band of diamond vibrations and absent in single-crystal graphite [46]. Meanwhile, the peak at 1580 cm^−1^ is associated with the G-band, which is broader in disordered graphite and incorporates the D′ band (on its high-frequency shoulder). Additionally, overtones (2D) and combination bands (D+D′) are observed [47]. The presence of carbyne is confirmed in both Type 1 and Type 2 coatings (Figure 3a and Figure 3b, respectively) by its distinctive wide band centered around 2100 cm^−1^ [42,48,49]. However, it is not possible to distinguish between contributions from the two carbyne configurations: polyynic chains [with alternating single-triple bonds (−C≡C−)_n_] at 2100 cm^−1^, and the less stable cumulenic chains [with double bonds (=C=C=)_n_], which are expected at 1980 cm^−1^ [41,42]. The relative carbyne/(D+G) signal ratio, i.e., the intensity ratio of the carbyne band to the sum of the D and G bands, is at least 2.3% for Type 1 and ≥1.7% for Type 2 coatings. Higher values are advocated when a constant background correction is applied (see Figure S3a,b) instead of a polynomial one. Background correction is problematic as there are no baseline regions without significant Raman peaks. For this reason, the XPS data discussed above are better suited to calculate the ratio of (sp^1^-coordinated) carbyne species to sp^2^- and sp^3^-bonded carbon components. Finally, the Raman microscopy images of both types of coatings (insets in Figure 3a,b) show uniform intensity on the micrometer scale, with variations over a larger field of view. Similar conclusions are drawn from the AFM data (Figure S4), with nanometer-scale root-mean-square (RMS) surface roughness measured on the micrometer scale, and larger RMS values observed for larger scan areas.

Contact angle measurements using water and diiodomethane were performed on the plain SiO_2_ surface, as well as on the carbyne-enriched Type 1 and Type 2 carbon coatings, to calculate the surface free energy values and their dispersion and polar components using the Owens–Wendt–Kaelble approach (Figure S5a). For comparison, these quantities were also calculated for the APTES monolayer [50] and amorphous [51] carbon (Figure S5a) based on the contact angle literature data. Additionally, the corresponding water contact angle (WCA) values are presented in Figure S5b. The results obtained for the surface energy, its components, and the water contact angle depend significantly on the chemical bonding and carbon hybridization sp^n^ states [51]. Specifically, oxidized surfaces form stronger dipole moments and stronger interactions with water as the presence of oxidized states increases the polar component of surface energy and improves wettability [52]. Therefore, the polar surface energy component and WCA values determined for the SiO_2_/Si surface (13.5 mJ/m^2^, 61°) can be attributed to the oxygen content, which is lower for oxygen–carbon bonds in the Type 1 (3.2 mJ/m^2^, 79.5°) and Type 2 coatings (3.6 mJ/m^2^, 75.2°). In comparison, the silicon surface modified with APTES is characterized by the polar surface energy component (5.9 mJ/m^2^) being higher and the water contact angle (65°) being lower than the corresponding values of both carbyne-enriched coatings. In turn, amorphous carbon yields a WCA value (77.6°) similar to that of Type 1 and Type 2 coatings but its polar SE component (11.1 mJ/m^2^) is much higher [51]. This can be related to a different ratio of sp^2^- and sp^3^-bonded carbons, reflected by the intensity ratio of the D and G peaks in the Raman spectra [51]. Finally, it has also been observed that surfaces rich in sp^3^-bonded carbon exhibit higher total surface energy and greater wettability compared to those rich in sp^2^-bonded carbon [53]. Therefore, the higher content of sp^3^-bonded carbon in Type 2 coatings (see Table S1) correlates with their surface energy and WCA values (44.2 mJ/m^2^, 75°) compared to those of Type 1 coatings (39.2 mJ/m^2^, 79.5°).

Rapid reaction with the ambient atmosphere of the carbyne species, produced during UHV carbon plasma deposition, results in carbyne-enriched carbon coatings Type 1 and Type 2 with the physicochemical properties described above. The performance of such coatings as efficient biosensor platforms in terms of immobilization of biomolecules will be analyzed in the following section. Before that, let us review their main features. First, the carbyne reaction with oxygen reduces the content of the sp^1^-bonded carbon to a small but non-zero value and introduces oxygen-rich carbon environments, both representative of Type 1 and Type 2 coatings. Second, these nearly hydrophobic coatings have a polar component of surface energy that is distinctly lower than that of amorphous carbon and oxidized or APTES-modified silicon. In addition, the water contact angle of these coatings is higher than that of APTES-modified silicon surfaces. The following chemical composition, carbon hybridization sp^n^ states distribution, and surface energy (SE) have been determined for Type 1 and Type 2 carbyne-enriched carbon coatings: Type 1: C_82_O_14_Si_2_Ar_2_ (3.9% sp^1^/48.6% sp^2^/47.5% sp^3^), SE 39.2 mJ/m^2^; Type 2: C_87_O_10_Si_2_Ar_1_ (2.9% sp^1^/45.7% sp^2^/51.4% sp^3^), SE 44.2 mJ/m^2^.

### 3.2. Biomolecule Immobilization onto Chips Modified with Carbyne-Enriched Carbon Layer

Chips modified with both types of carbyne-enriched carbon layers were evaluated as biosensing surfaces through the immobilization of biomolecules either by covalent bonding or by physical adsorption. The former was based on the observation of peaks corresponding to C=O in the C1s core-level XPS spectra (Figure 2) that might indicate the presence of aldehyde- or carboxy-groups. Thus, chips modified with Type 1 and Type 2 carbyne-enriched carbon layers were reacted with an amine derivative of biotin after activation with an EDC/NHS mixture, followed by probing with streptavidin. For the physical adsorption, an antibody against CRP was immobilized and the surfaces were evaluated through a two-step non-competitive immunoassay for CRP. In the first case, SiO_2_/Si chips without any modification were used as control surfaces, whereas in the second case, chips modified with APTES were employed.

#### 3.2.1. Covalent Bonding of Amine Derivative of Biotin

**Figure 4:**
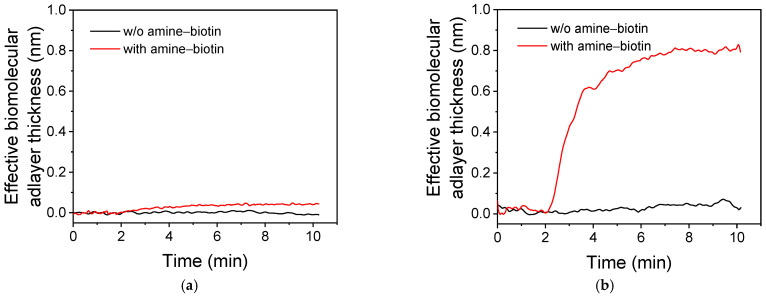
Real-time responses obtained upon running a 5 μg/mL streptavidin solution over (**a**) an unmodified SiO_2_/Si chip or (**b**) a SiO_2_/Si chip modified with carbyne-enriched carbon coating Type 1. Both chips have been treated with an EDC/NHS solution without (black line) and with subsequent reaction with an amine–biotin derivative (red line).

The real-time responses obtained upon running a streptavidin solution from an unmodified chip SiO_2_/Si (a) or a SiO_2_/Si chip modified with a carbyne-enriched carbon coating (b)—both of which have been reacted with EDC/NHS and then with an amine derivative of biotin—are presented in Figure 4. The responses obtained from surfaces, which were activated with EDC/NHS but not reacted with the amine–biotin derivative prior to running the streptavidin solution, are also provided in the respective graphs as blanks (black lines in Figure 4a,b). As shown, the chip with the carbyne-enriched carbon coating provided considerable signal upon running the streptavidin after activation with EDC/NHS and reaction with the amine–biotin derivative, whereas without reaction with the amine–biotin derivative the signal was negligible. The response obtained from the chips with the carbyne-enriched carbon layers indicates the presence of carboxy groups, which are converted into active ester groups upon treatment of the surface with the EDC/NHS mixture, enabling the binding of the amine–biotin derivative. It should be noted that there were no statistically important differences in the responses obtained from chips modified with the two types of carbyne-enriched carbon coatings.

On the other hand, the unmodified SiO_2_/Si provided a low response upon probing with streptavidin, which was, however, clearly distinguished from the blank. The response observed from the unmodified SiO_2_/Si surfaces could be attributed to the reaction of EDC/NHS and then with the amine–biotin derivative with the silanol groups created on the surface during cleaning with Piranha solution [54].

The biotin–streptavidin binding assay was used as a proof-of-concept, similarly to the previous literature [55,56], to demonstrate the covalent bonding capabilities of chips modified with a carbyne-enriched carbon coating. This supports their potential application in covalently attaching low-molecular-weight recognition molecules, such as haptens, aptamers, and others. These modified chips could then be employed in competitive immunoassays for the detection of haptens [56]. Furthermore, modifying the sensor surface with biotin enables the indirect binding of recognition molecules, which can be advantageous over direct binding as it helps preserve the functional properties of the immobilized recognition elements [57].

#### 3.2.2. Physical Adsorption of Anti-CRP Antibody

**Figure 5:**
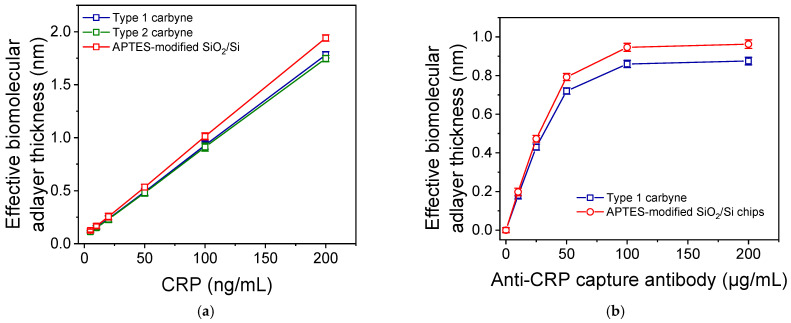
(**a**) CRP calibration curves obtained from SiO_2_/Si chips with Type 1 (blue line) and Type 2 (green line) carbyne-enriched carbon layers as well as from APTES-modified SiO_2_/Si chips (red line). (**b**) Signal values obtained from SiO_2_/Si chips with Type 1 (blue line) carbyne-enriched carbon layer and APTES-modified SiO_2_/Si chips (red line) coated with capture anti-CRP antibody at concentrations ranging from 10 to 200 μg/mL for CRP calibrator of 100 ng/mL. Each point is the average of three measurements ± SD.

The two types of chips with a carbyne-enriched carbon layer were compared with the APTES-modified SiO_2_/Si chips, the standard substrates used in WLRS biosensing technology, in terms of the signals provided for different CRP calibrators after immobilization of an anti-CRP antibody through physical adsorption. Figure 5a presents the calibration curves derived from the three types of chips. As shown, the analytical signal values obtained from the standard APTES-modified SiO_2_/Si chips were approximately 10% higher compared to those from chips with a carbyne-enriched carbon layer. On the other hand, the signals obtained from the two types of chips with a carbyne-enriched carbon layer were almost indistinguishable. However, the detection sensitivity and dynamic range for all three substrates were found to be identical. Specifically, the detection limit (LOD) of the assay, calculated as the concentration corresponding to +3SD of 10 replicate measurements of the zero calibrator, was 2.0 ng/mL for all three types of chips. Similarly, the limit of quantification (LOQ), defined as the concentration corresponding to +6SD of 10 replicate measurements of the zero calibrator, was 5 ng/mL for all chips.

Chips modified with the Type 1 carbyne-enriched carbon layer were also compared with APTES-modified SiO_2_/Si chips regarding the concentration of affinity-purified goat polyclonal anti-CRP antibodies that provided the maximum plateau signal values. The results, shown in Figure 5b, demonstrate that the analytical signal increased with higher concentrations of immobilized antibodies, reaching maximum values at concentrations of 100 μg/mL or higher for both substrates.

### 3.3. Stability of Biomolecule Binding Properties of Chips with Carbyne-Enriched Carbon Layers

Another aspect in which the SiO_2_/Si chips with a carbyne-enriched carbon layer were compared to APTES-modified ones was the stability of their biomolecule binding capacity over time. Both types of chips were stored at room temperature in a desiccator. To assess stability, different chips from the same batch were tested over a period of 18 months.

**Figure 6:**
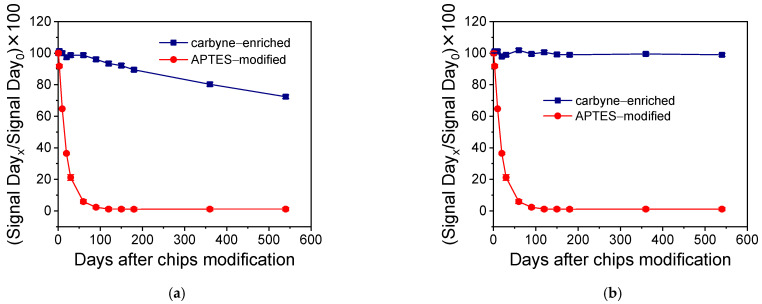
Percent signal values obtained in the span of 540 days with respect to the value obtained the first day after their preparation from SiO_2_/Si chips modified with carbyne-enriched carbon layers (squares) or APTES (circles) chips, on which, (**a**) an amine–biotin derivative has been covalently bound or (**b**) an anti-CRP antibody was physically adsorbed and then assayed with a 100 ng/mL CRP calibrator. Each value is the average of three chips ± SD.

For comparison of the stability of the surfaces for biomolecules binding through covalent bonding, the chips with a carbyne-enriched carbon coating were assessed following the procedure described in Section 3.2.1, whereas the amine groups of the APTES-modified chips were transformed to active esters via reaction with a bi-functional NHS-ester (BS3) to enable binding of the amine–biotin derivative. In Figure S6, the real-time response obtained upon running a streptavidin solution over an APTES-modified chip after reaction with BS3 and the amine–biotin derivative is indicatively provided. In Figure 6a, the percent signal values are presented, obtained over a period of 18 months (540 days) with respect to the value obtained the first day after their preparation, from SiO_2_/Si chips modified with a carbyne-enriched carbon layer (squares) or APTES (circles), on which an amine–biotin derivative has been covalently bound. As shown, the APTES-modified chips lost more than 90% of their initial binding capacity 2 months after their preparation, while the chips with the carbyne-enriched carbon coating kept almost 70% of their initial binding capacity after 18 months.

Regarding the comparison in terms of biomolecule immobilization through physical adsorption, chips modified with a carbyne-enriched carbon coating and APTES were used without further modification. The immobilization of anti-CRP antibodies was performed immediately before the assay, and a 100 ng/mL CRP calibrator was used for the test. The respective percent signal values obtained over a period of 18 months from surfaces modified either with a carbyne-enriched carbon layer or APTES are depicted in Figure 6b. As shown, the APTES-modified chips have lost almost 95% of their initial protein adsorption capacity after two months, whereas the chips with the carbyne-enriched carbon coating preserved their protein adsorption capacity for 18 months. This is the most significant advantage of carbyne-enriched carbon coatings compared to chemically activated SiO_2_/Si, such as APTES-modified chips, and supports the use of chips with a carbyne-enriched carbon layer not only in the WLRS platform but also in other optical biosensors based on similar transduction principles or materials.

## 4. Conclusions

This study presents the successful deposition through arc-pulse carbon plasma of carbyne-enriched carbon layers on SiO_2_/Si chips as well as their physicochemical characterization and evaluation as biosensing substrates for the WLRS sensing platform. The presence of carbyne in the carbon coatings formed on the silicon chips was confirmed using spectroscopic and surface imaging techniques, which also defined the composition of the layer, showing variations in bond contributions and carbon hybridization states depending on the film deposition conditions. However, these differences did not have an impact on the performance of the chips with carbyne-enriched carbon layers when used as substrates for the immobilization of biomolecules, either through covalent bonding or physical adsorption. It was also found that the SiO_2_/Si chips with the carbyne-enriched carbon layer exhibited very similar biomolecule binding capacity as the standard APTES-modified SiO_2_/Si chips used in WLRS technology for both covalent and physical immobilization approaches. Although the proposed surface modification did not enhance the analytical performance of the specific sensor, the chips with carbyne-enriched carbon layers demonstrated remarkable long-term stability. They retained approximately 70% and over 95% of their initial biomolecule immobilization capacity via covalent bonding and physical adsorption, respectively, over an 18-month period. In contrast, the APTES-modified chips rapidly lost their biomolecule binding capabilities. This stability is a critical advantage of the proposed surface modification, especially for transitioning biosensors from laboratory research to commercial applications. It is expected to simplify chemical activation and biofunctionalization processes. Furthermore, the proposed modification supports biomolecule immobilization through both physical adsorption and covalent bonding, making it compatible with a wide range of biorecognition elements. This versatility is particularly valuable given the diversity of silicon-based transducers, such as optical fibers, integrated interferometers, ring resonators, and reflectometric sensors, all of which require chemical activation before attaching specific biorecognition elements. As a result, substrates with carbyne-enriched carbon layers hold significant potential for broader use in optical biosensors and related technologies.

## Data Availability

The original contributions presented in this study are included in the article/Supplementary Materials. Further inquiries can be directed to the corresponding authors.

## Supplementary Materials

The following supporting information can be downloaded at https://www.mdpi.com/article/10.3390/nano15181384/s1, Figure S1: (a) Image of the WLRS-based biosensor instrument used in this study. (b) Schematic of the two-site immunoassay for CRP determination using the carbyne-modified SiO_2_/Si chips, including the following: (1) incubation of the immobilized onto the carbyne-modified chip anti-CRP antibody with the CRP calibrator/sample; (2) reaction of the bound CRP to chip immobilized antibody molecules with the anti-CRP antibody to form the “sandwich”-like immunocomplexes; and (3) biochip surface regeneration, i.e., removal of the immunoadsorbed molecules to use the chip for a new assay cycle; Table S1: Fractions of carbon hybridization types revealed by XPS in this and other works [29,30,31,32]. Uncertainty is described by the last digit of the result; Figure S2: XPS composition of the surface and interior of Type 1 and Type 2 carbyne-enriched carbon coatings compared to that of the reference SiO_2_/Si chip surface (denoted as SiO_2_). Atomic concentrations (%) of carbon with different hybridization, elements in functional groups, and other elements are marked with different symbols. The error bars are smaller than the symbols used; Figure S3: Raman micro-spectroscopic analysis of (A) Type 1 and (B) Type 2 carbyne-enriched coatings presented in Figure 3a and Figure 3b, respectively. A constant background correction is applied instead of a polynomial one (used for Figure 3a,b). Background correction is problematic as there are no baseline regions without significant Raman peaks; Figure S4: AFM images with root-mean-square RMS surface roughness values obtained for different scan areas (left 10 × 10 μm^2^; right 2 × 2 μm^2^) of Type 1 (a) and Type 2 (b) carbyne coatings; Figure S5: (a) Stacked bar chart representing surface free energy (overall bar heights) and its dispersion and polar components (blue and green sub-bars, respectively) calculated with the Owens–Wendt–Kaelble approach based on contact angle data for water and diiodomethane for plain SiO_2_/Si surface, Type 1 and Type 2 carbyne coatings, along with the literature values for hydrolyzed silicon surface modified with APTES [53] and amorphous carbon (a-C) [51]. (b) Water contact angle (WCA) values for the same surfaces; Figure S6: Real-time response obtained upon running a 5 μg/mL streptavidin solution over a SiO_2_/Si chips modified with APTES after reaction with BS3 and an amine–biotin derivative.
