# Supplementary material for: Carbyne-Enriched Carbon Coatings on Silicon Chips as Biosensing Surfaces with Stable-over-Time Biomolecule Binding Capacity
Source: Nanomaterials (Basel). 2025 Sep 9;15(18):1384. doi: 10.3390/nano15181384 (PMC12472631; doi:10.3390/nano15181384)
Supplement: Supplementary file 1 [file nanomaterials-15-01384-s001.zip › nanomaterials-3845548-supplementary.pdf]

Calculation of "effective biomolecular adlayer thickness"

The WLRS reader is accompanied by a specially developed software which can evaluate the initial thickness of the SiO_2/biomolecular adlayer and transform the shift of the interference spectrum into the effective thickness of the biomolecular adlayer (nm), which is actually the signal of the WLRS sensor. More specifically, a reference [Ref(λ)] and a dark spectrum [D(λ)] are recorded prior to real-time continuous recording of the reflectance spectrum [S(λ)], and the absolute reflectance spectrum is calculated by equation (1):

$$R(\lambda) = S(\lambda) - D(\lambda)/(Ref(\lambda) - D(\lambda)) \qquad (1)$$

The normalized spectrum is further processed through the Levenberg–Marquart algorithm to calculate the thickness of the biomolecular adlayer, d_1, from the shift in the interference spectrum wavelength, δλ, according to Equation (2):

$$\delta\lambda = r_1 \times [1 - r_2 2/(r_1 + r_2) \times (1 + r_1 \times r_2)] \times (n_1 \times d_1)/(n_2 \times d_2) \times \lambda_{0m} \qquad (2)$$

where r_1 and r_2 and n1 and n_2 are the Fresnel coefficients and refractive indices of the biomolecular and the SiO_2 layer, respectively, d_1 and d_2 are the thickness of the two layers, and λ is the wavelength, where λ_{0m} is the reflectance extremum.

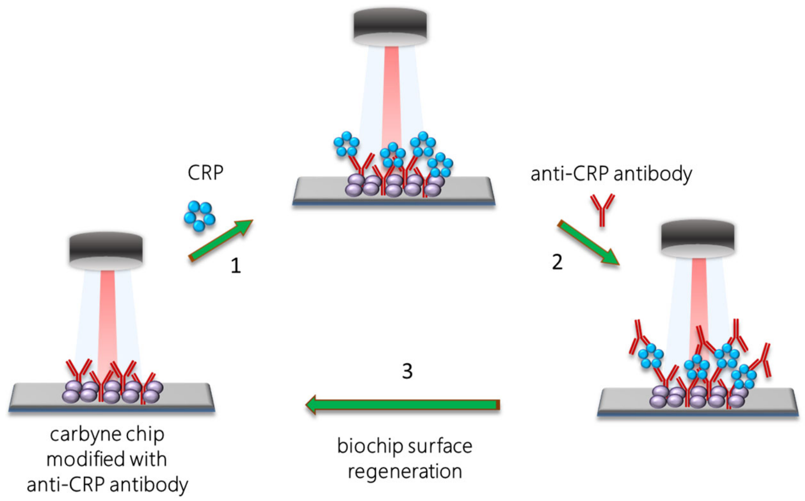

Figure S1. Schematic of the two-site immunoassay for CRP determination using the SiO_2/Si chips with the carbyne-enriched carbon coating including: (1) incubation of the immobilized onto the carbyne-modified chip anti-CRP antibody with the CRP calibrator/sample, (2) reaction of the bound CRP to chip immobilized antibody molecules with the anti-CRP antibody to form the “sandwich” like immunocomplexes, and (3) biochip surface regeneration, i.e., removal of the immunoadsorbed molecules to use the chip for a new assay cycle.

Table S1. Fractions of carbon hybridization types revealed by XPS in this and other works [31, 33,34,35]. Uncertainty is described by the last digit of the result.

**Sample**	**sp^1 (%)**	**sp^2 (%)**	**sp^3 (%)**
Type 1 (surface)	3.9	48.6	47.5
Type 2 (surface)	2.9	45.7	51.4
sp-based material (poly(vinylidene chloride-vinyl chloride) copolymer dehydrohalogenation) [30]	3.1	69.1	27.7
carbyne-rich tetrahedral carbon films on Si (fs pulsed laser ablation) [29]	~5	~40	~55
linear chained carbon 200 nm thick films on p-Si (carbon plasma) [31]	5.1	42.3	52.6
carbyne-containing 60 nm thick C films on Cu (rf magnetron sputtering) [32]	5.2	60.3	18.0

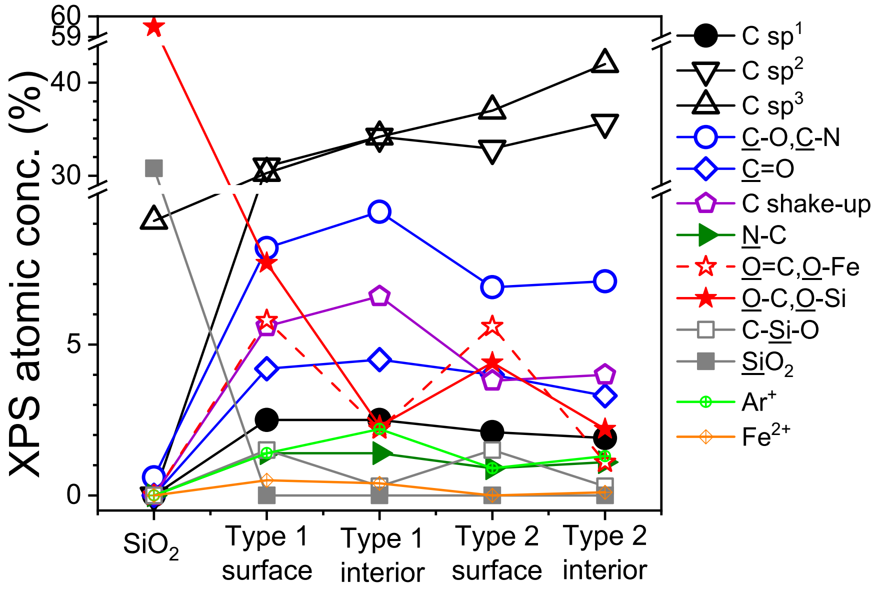

Figure S2. XPS composition of the surface and interior of Type 1 and Type 2 carbyne-enriched carbon coatings compared to that of the reference SiO_2/Si chip surface (denoted as SiO_2). Atomic concentrations (%) of carbon with different hybridization, elements in functional groups, and other elements are marked with different symbols. The error bars are smaller than the symbols used.

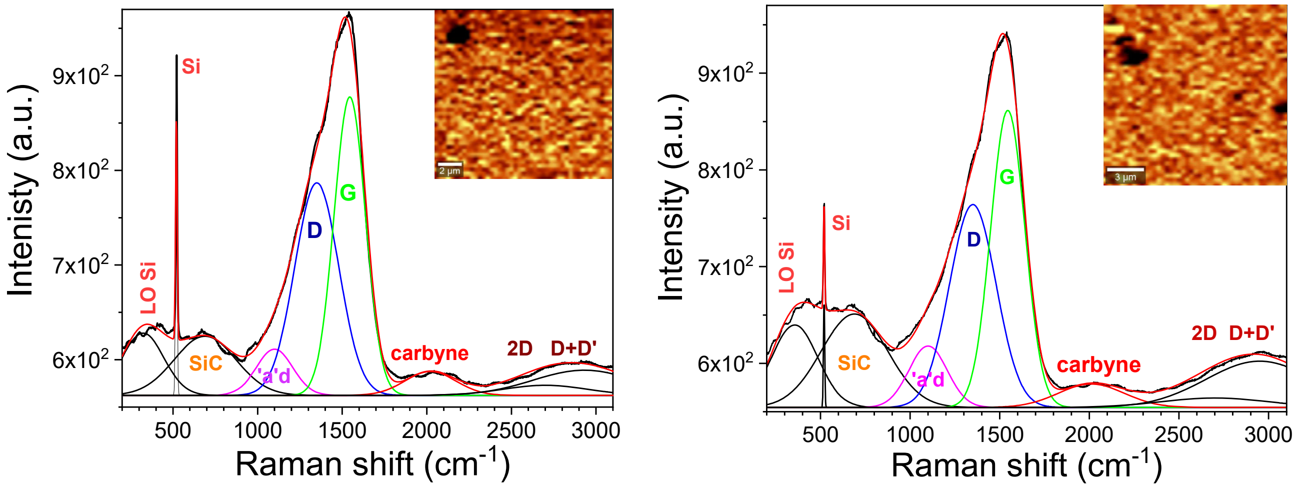

(a) (b)

Figure S3. Raman micro-spectroscopic analysis of (a) Type 1 and (b) Type 2 carbyne-enriched coatings presented in Figs. 3a and 3b, respectively. A constant background correction is applied instead of a polynomial one (used for Figs. 3a and 3b). Background correction is problematic as there are no baseline regions without significant Raman peaks.

(a)

(b)

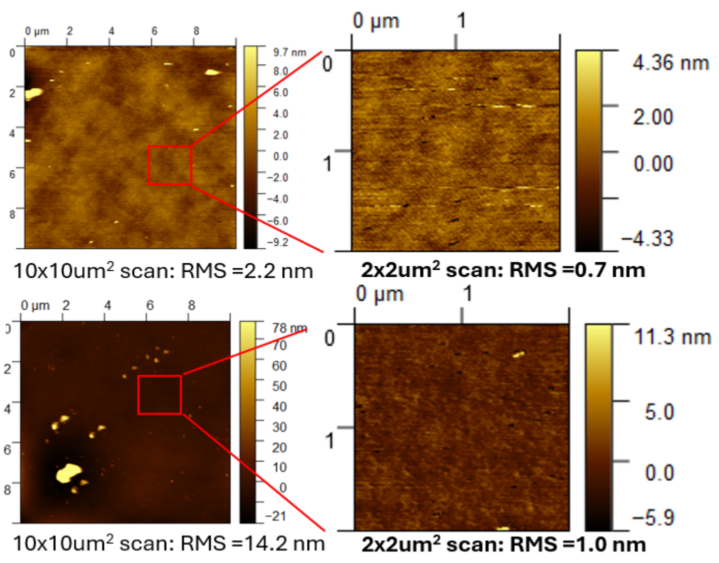

Figure S4. AFM images with root-mean-square RMS surface roughness values obtained for different scan areas (left 10x10 μm^2; right 2x2 μm^2,) of Type 1 (a) and Type 2 (b) carbyne coatings.

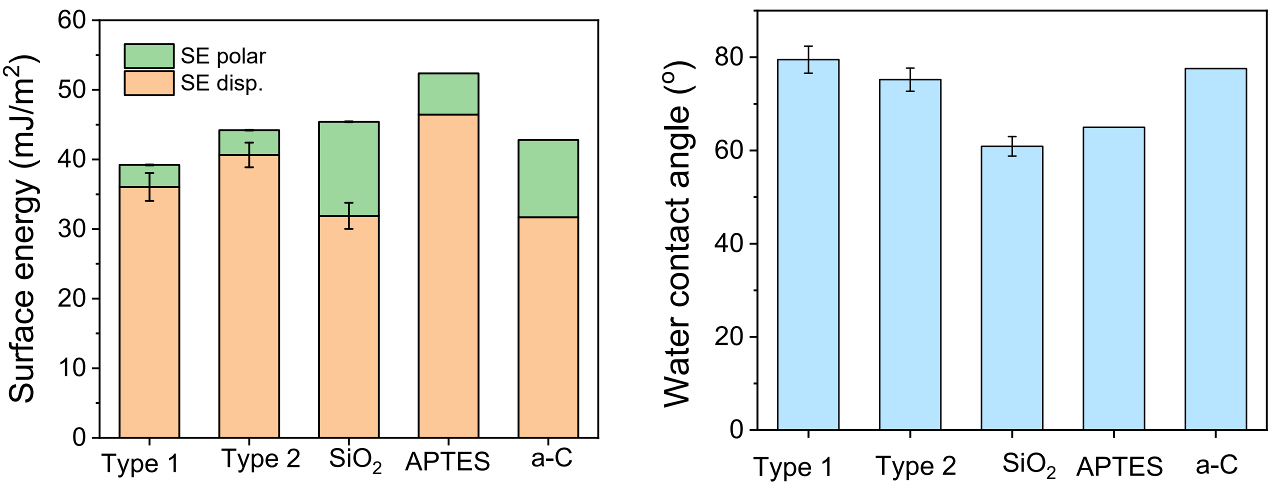

(a) (b)

Figure S5. (a) Stacked bar chart representing surface free energy (overall bar heights) and its dispersion and polar components (camel and green sub-bars, respectively) calculated with the Owens-Wendt-Kealble approach based on contact angle data for water and diiodomethane for plain SiO_2/Si surface, Type 1 and Type 2 carbyne coatings, along with literature values for hydrolyzed silicon surface modified with APTES [53] and amorphous carbon (a-C) [51]. (b) Water contact angle (WCA) values for the same surfaces.

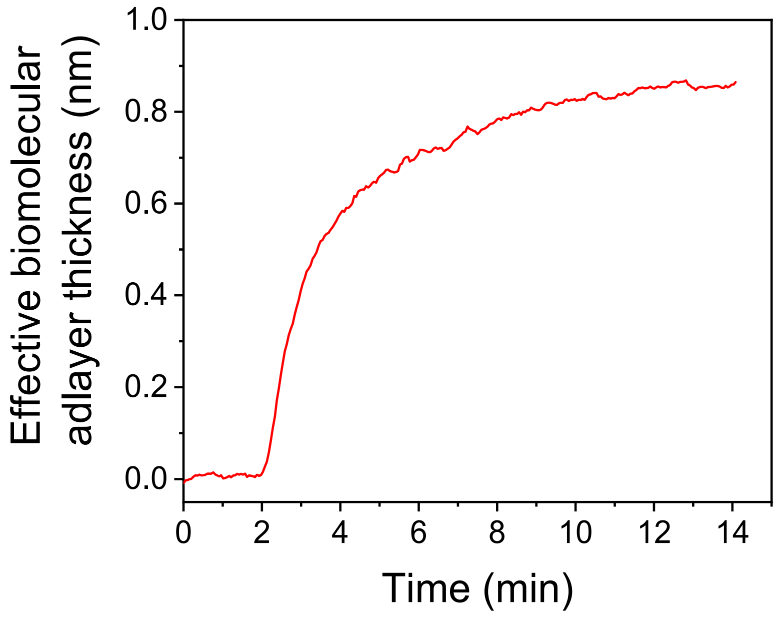

Figure S6. Real-time response obtained upon running a 5 µg/mL streptavidin solution over a SiO_2/Si chips modified with APTES after reaction with BS3 and an amine-biotin derivative.
